# Genome-wide co-expression analysis predicts protein kinases as important regulators of phosphate deficiency-induced root hair remodeling in Arabidopsis

**DOI:** 10.1186/1471-2164-14-210

**Published:** 2013-04-01

**Authors:** Ping Lan, Wenfeng Li, Wolfgang Schmidt

**Affiliations:** 1 State Key Laboratory of Soil and Sustainable Agriculture,Institute of Soil Science, Chinese Academy Sciences, Nanjing, 210008, People’s Republic of China; 2 Institute of Plant and Microbial Biology, Academia Sinica, Taipei 115, Taiwan

**Keywords:** Protein phosphorylation, RNA sequencing, Co-expression analysis, Root hairs, Phosphate deficiency

## Abstract

**Background:**

Phosphorus (P) is one of the essential but often limiting elements for plants. Based on transcriptional profiling we reported previously that more than 3,000 genes are differentially expressed between phosphate (Pi)-deficient and Pi-sufficient Arabidopsis roots (MCP 11(11):1156–1166, 2012). The current study extends these findings by focusing on the analysis of genes that encode protein kinases (PK) and phosphatases (PP) by mining PK and PP genes that were differentially expressed in response to Pi deficiency.

**Results:**

Subsets of 1,118 and 205 annotated PK and PP genes were mined on the basis of the TAIR10 release of the Arabidopsis genome. Analysis of RNA-seq data showed that 92 PK and 19 PP genes were not detected in roots (zero reads in three biological repeats); 96 PK and 10 PP showed low abundance (≤ 10 reads). Gene ontology analysis revealed that the 188 PK genes with no or low expression level in Arabidopsis roots are mainly involved in pollen recognition, pollen tube growth or other processes not relevant for root hair formation. More than 50% of the cysteine-rich RLK (receptor-like protein kinase) subfamily genes belong to this group. Among the 29 PP genes with no or low expression level, purple acid phosphatases, haloacid dehalogenase-like hydrolases, and PP2C genes with functions in the dephosphorylation of RNA polymerase II C-terminal domain and mRNA capping were enriched. Subsets of 173 PK and 35 PP genes were differentially expressed under Pi-deficient conditions. Putative functional modules (clusters) of these PK and PP genes were constructed based on co-expression analysis using the MACCU toolbox. A co-expression network comprising 65 known or annotated PK and PP genes (60 PK and 5 PP genes, respectively) was subdivided into several highly co-expressed gene sub-clusters. The largest sub-cluster was composed of 22 genes, most of which have been assigned to the RLK superfamily and were associated with cell wall metabolism, pollen tube and/or root hair development and growth.

**Conclusions:**

We here provide comprehensive ‘digital’ transcriptional information on PK and PP genes in Arabidopsis roots. The co-expression network derived from our data mining approach sets the stage for follow-up experimentation that helps to complete our understanding of the post-translational regulation of Pi deficiency-induced changes in root hair morphogenesis.

## Background

Phosphorous, mainly taken up as phosphate (Pi) by plants, is an essential micronutrient involved in signaling, metabolism and photosynthesis. The bioavailability of Pi is often very low due to its tendency to form complexes with soil cations. In agricultural systems, Pi deficiency is a major cause of severe yield losses in crops and poor quality of edible plant parts. Low Pi availability is often corrected by the application of large quantities of fertilizers, which is associated with environmental pollution and substantial costs. Understanding how plants adapt to low Pi availability is thus mandatory to develop Pi-efficient germplasms. To cope with low Pi availability, plants have evolved an array of adaptive processes aimed at improving Pi uptake and re-mobilization, comprising the acquisition and redistribution of Pi, alterations in developmental programs, and metabolic networks [1]. Proteomic [2-4] and transcriptomic [5-11] profiling studies have uncovered several robustly changed processes in Pi-deficient plants, including the remodeling of lipid metabolism, changes in glycolytic carbon flux, alterations in root development, and rewired signaling pathways [12-14].

The mechanisms underlying the maintenance and recalibration of cellular Pi homeostasis are complex. The Myb-type transcription factor PHOSPHATE STARVATION RESPONSE1 (PHR1) is a central conserved regulator that controls a subset of Pi deficiency genes by binding to an imperfect palindromic sequence motif [15,16]. Consistent with a critical regulatory role of PHR1 in Pi homeostasis, overexpression of *PHR1* led to increased Pi accumulation [17]. The activity of PHR1 is controlled by the SUMO E3 protein ligase SIZ1 [18], representing the most upstream component of the Pi deficiency signaling cascade identified so far. Another subset of Pi-responsive genes is regulated by the E2 ubiquitin conjugase PHOSPHATE2 (PHO2). A connection between these two central switches is established by MicroRNA399 (MiR399), which systemically controls PHO2 through transcript cleavage [19,20]. MiR399 itself is strongly induced by Pi deficiency [8]. The sensor for Pi remains to be discovered.

Besides the involvement of protein ubiquitination [18,21], other posttranslational processes potentially involved in the Pi deficiency response have not been thoroughly investigated. An estimated one-third of all eukaryotic proteins undergoes reversible phosphorylation via protein kinases (PK) and phosphatases (PP), demonstrating the importance of this process. Modifications of protein with phosphate can affect protein structure, activity, localization, interaction, and stability, thereby regulating crucial processes such as metabolism and development. Several hundred genes encoding PKs and PPs were found to be differentially expressed upon Pi deficiency by transcriptional profiling of roots from Pi-deficient plants [22], suggesting that alterations in protein phosphorylation patterns induced by Pi deficiency are critical in the control of Pi homeostasis. For example, under Pi-limiting conditions the high-affinity phosphate transporter*PHT1;1* was found to be induced and newly–synthesized PHT1;1 protein was phosphorylated by an yet unknown PK at the C-terminal 514 amino acid Ser, which is required for the precise localization of PHT1;1 to the plasma membrane [13].

Transcriptome analysis alone, however, is insufficient for defining potential roles of differentially expressed PKs and PPs genes in Pi homeostasis. Functional characterization of these genes by reverse genetic approaches such as increasing or decreasing their transcript level (by T-DNA insertion and/or RNAi) is required to elucidate their biological functions. However, individually assaying hundreds to thousands of differentially expressed genes without any selection filter would be extremely laborious. Systemic cluster analyses provide a means to filter and select genes of interest for the biological question addressed. Genes showing similar expression pattern under diverse conditions often have correlative functions [23], and the biological processes in which genes with unknown functions are involved can be predicted based on co-expression data (‘guilt by association’) [24,25].

In the present study, the global expression of PK and PP genes in Arabidopsis roots was analyzed in order to gain insights into the regulation of the interplay of transcriptional and post-translational responses to Pi deficiency. By mining public databases, PK and PP genes that are differentially expressed upon Pi starvation were clustered into groups of closely correlated modules based on their co-expression under various sets of experimental conditions. Using this approach, we discovered several potentially critical regulatory PKs with roles in root hair development and growth under Pi deficiency.

## Results and discussion

### Digital information on the expression of protein kinase and phosphatase genes in Arabidopsis roots

**Figure 1:**
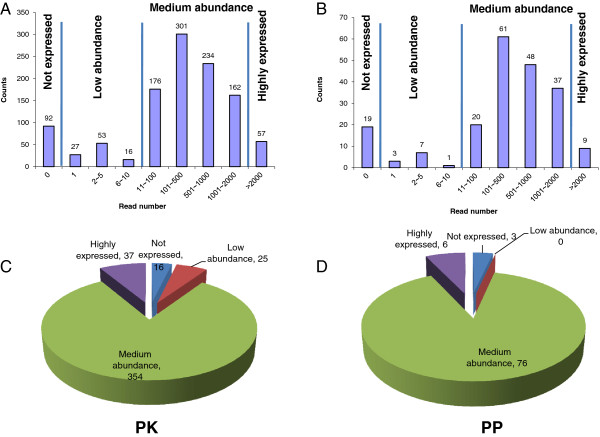
**The PK and PP transcriptome and proteome in Arabidopsis roots.** (**A**) Number and expression levels of PK genes. (**B**) Number and expression levels of PP genes. (**C**) Number of PK proteins identified at different gene transcription level. (**D**) Number of PP proteins identified at different gene transcription level.

Protein kinases and phosphoylases play key roles in regulating nearly all aspects of cellular processes. However, due to the use of microarray probe sets that have significant cross hybridization potential and are unable to distinguish highly similar genes of this subfamily, transcriptional information on the expression of PK and PP genes is incomplete in Arabidopsis. In addition, tissue-specific gene expression information of PKs and PPs is lacking. The RNA-seq technology has proven to provide precise digital information on gene expression, and is able to discriminate genes of high sequence identity [26]. Using this technology, we previously examined global gene expression changes upon Pi deficiency in Arabidopsis roots [22]. Focusing on the expression of PKs and PPs, we mined and re-analyzed our RNA-seq data set (NCBI: SRA050356.1). Based on the PK and PP gene families annotated in the TAIR10 release of Arabidopsis genome, a total of 1,118 PK (GO: 0004672, Additional file 1) and 205 PP (GO: 0004721, Additional file 2) genes was retrieved and compared with the RNA-seq data. We defined a gene as not being expressed if the unique read number was zero in all three biological repeats under normal (Pi-replete) conditions; low abundance of a transcript was defined by a unique read number ≤ 10 in either of the three biological repeats. A gene was defined as being highly expressed in Arabidopsis roots when the read number was higher than 2,000 in either of the three biological repeats. On the basis of these criteria, 92 PK genes were not detected in Arabidopsis roots, transcripts of 96 PK genes were low abundant; and 57 PK genes were highly expressed (Figure 1A and Additional file 3). For the 1,118 *PK* genes, 432 cognate proteins were identified with at least one uniquely matched peptide ([22] and Additional file 4). Generally, PK proteins were more likely detected when the associated transcript was highly abundant. However, proteins were also detected from about 20% of the genes with low abundant or absent transcripts (Figure 1C and Additional file 4) confirming the observation that gene expression is not always correlated with protein abundance [22].

**Figure 2:**
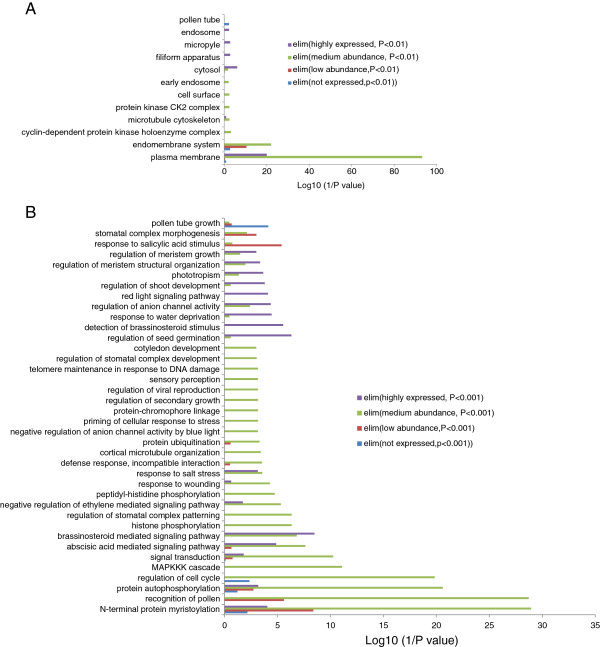
**Gene ontology (GO) enrichment analysis of four types of PK genes.** (**A**) Subcellular localization of the four types of PK genes. (**B**) Biological process of the four types of PK genes.

Differential Gene Ontology (GO) enrichment analysis revealed that the products of most PK genes in Arabidopsis roots are chiefly localized in the endomembrane system and plasma membrane, especially for proteins encoded by medium and highly expressed PK genes(Figure 2A, P < 0.01), and are involved in diverse biological processes. The largest amount of genes with medium abundance was involved in several biological processes, with the GO categories ‘recognition of pollen’, ‘regulation of cell cycle’, and ‘signal transduction’ being most enriched (Figure 2B, P < 0.01). The biological processes ‘recognition of pollen’, ‘stomatal complex morphogenesis’, and ‘response to salicylic acid stimulus’ were overrepresented in low abundant genes or in genes that were defined as not being expressed (Figure 2B, P < 0.01). In particular, more than 50% of genes from the cysteine-rich RLK (receptor-like protein kinase) subfamily were not or lowly expressed in Arabidopsis roots (Additional file 5). Cysteine-rich RLKs are reportedly involved in pathogen response, ABA signaling and are regulated by ozone and hormones [27-29]. Two genes of this family, *CRK19* and *CRK29*, that phylogenetically belong to different groups, were highly expressed in Arabidopsis roots (but not regulated by Pi deficiency; Additional file 5), suggesting that these two CRKs may be critical for root development. Unfortunately, we did not detect the corresponding proteins by uniquely matched peptides in our proteomic study [1]; thus, the possibility that post-transcriptional processes regulate the abundance of these proteins cannot be ruled out. For the 57 high abundant PK genes, GO enrichment analysis showed that those genes whose products are localized in the plasma membrane, the cytosol, or the filiform apparatus were highly enriched (Figure 2A and Additional file 6). This result is consistent with the fact that most of these proteins belong either to the leucine-rich repeat receptor-like protein kinases (LRR-RLK) which often contain a trans-membrane domain, or are calcium-dependent protein kinase (CDPK)-SNF1-related kinases (SnRK), which are mostly cytosolic enzymes. Several biological processes within the GO category ‘response to environmental stimuli’ including ‘water deprivation’ and ‘salt stress’ among others, as well as responses to hormones such as ABA, cytokinin, and brassinosteroid were enriched (Figure 2B and Additional file 6), indicating that roots are subjected and respond to external and internal stimuli during growth and development even under artificial growth conditions in the laboratory. Unexpectedly, the biological processes ‘response to light’ and ‘response to red light’ were also enriched. One possible explanation is that these genes are auxin-related, and the proper localization of PIN auxin transporters is dependent on phytochromes [30].

Applying the same criteria used for the PK genes, subsets of 19 and 10 PP genes were not detected or lowly expressed in Arabidopsis roots, respectively (Additional file 7). The trend of PP protein expression was similar to that of PK proteins (Figure 1D and Additional file 4). Among these two subsets, purple acid phosphatases (PAP), haloacid dehalogenase-like hydrolases (HAD), and PP2C genes were enriched, with gene products mainly localized in nucleus and functioning in the biological processes ‘dephosphorylation of RNA polymerase II C-terminal domain’ and ‘mRNA capping’ (Additional file 8). Products of the nine highly expressed PP genes (Additional file 7) were mainly involved in the formation of PP-1 and PP2A complexes associated with the biological processes ‘embryonic root morphogenesis’ and ‘phosphate ion homeostasis’ (Additional file 9). The lack of a transcriptional response of PP genes to Pi deficiency suggest that under normal conditions in Arabidopsis roots cellular Pi homeostasis is regulated by PPs, probably by protein de-phosphorylation. Uncovering the substrates regulated by these PP genes would strongly facilitate our understanding of cellular Pi homeostasis.

### Identification of Pi-responsive genes encoding protein kinase and phosphatase in Arabidopsis roots

Although approximately one thousand genes have been identified as being Pi-responsive by using the full-genome Affymetric ATH1 gene chip [5-11], the technical limitations of the microarray technology renders a precise estimation of the changes in the expression of Pi-responsive genes difficult. Using RNA-seq, we defined a total of 3,106 genes as differentially expressed between Pi-sufficient and Pi-deficient Arabidopsis roots [22]. Subsets of 173 PK and 35 PP genes, comprising diverse subfamilies were differentially expressed under Pi-deficient conditions (Additional file 10), some of which have been listed as Pi-responsive genes in earlier microarray studies [5-11]. Among the differentially expressed PK genes, members of the RLK superfamily genes, especially from the LRR-RLK subfamily, and genes from the CDPK-SnRK superfamily were enriched, while members of the PAP and PP-2C families were predominant in the differentially expressed genes encoding PPs. In total, 205 PK and PP genes were finally mined for further analysis. Among them, three genes (At1g49580, At2g01830 and At2g20050) are annotated as harboring both PK and PP activity. Gene ontology analysis revealed that those genes whose products are localized on the plasma membrane, the cell surface, the ER, the cell wall, or are associated with the ubiquitin ligase and calcineurin complexes were highly enriched (P < 0.01; Additional file 11). Purple acid phosphatases, members of the largest class of plant acid phosphatases, are generally assumed to be involved in intra-and/or extra-cellular Pi scavenging and recycling of Pi under Pi-deficient conditions. In Arabidopsis, the PAP family is composed of 29 members sharing conserved domains. Most *PAPs* are induced by Pi deficiency, some of which in an organ-specific manner [31,32]. Precise digital expression information of the 29 members of the PAP family in Pi-deficient Arabidopsis rootsis presented by RNA-seq (Additional file 12), which completes gene expression information in Arabidopsis roots so far uncovered by microarray analysis and classic molecular techniques. It is unclear, however, whether the members of the PAP family harbor PP catalytic activity.

### Construction of Pi-responsive PK and PP co-expression networks

Co-expression networks of the differentially expressed PK and PP genes were constructed using the MACCU software [9]. Co-expressed genes were selected with a Pearson correlation coefficient cutoff of 0.7. This cutoff also has been used in earlier studies [9,33]. Co-expression networks constructed with this cutoff are well suited to guide follow-up experiments (i.e. networksare neither too big nor too small). It should be mentioned that the co-expression network constructed here is restricted to roots and the 300 public microarrays mined for generating co-expression relationship were root-related experiments [9]. Because protein regulation by phosphorylation is reversible and requires both PKs and PPs, the network was constructed from both PK and PP genes. The 205 differentially expressed PK and PP genes were loaded as guide genes to calculate the correlations. Correlations between guide genes were visualized by Cytoscape (http://www.cytoscape.org). In the co-expression network, a node represents a gene and an edge represents the correlation between two genes. The network of PK and PP genes responsive to Pi deficiency consists of 65 nodes and 96 edges (Additional file 13). The 65 nodes contain 60 genes that encode PKs and five genes that encode PPs (Additional file 14). The network can further be divided into two larger and seven smaller modules. Genes with similar expression pattern under diverse conditions can have correlative functions and may form a functional module [23]. Only 34% of the input genes were constituents of the co-expression network, suggesting that the majority of PKs and PPs responsive to Pi deficiency are functionally diverse and involved in a variety of biological processes and metabolic pathways. It is noteworthy that, compared to14% of the input PP genes associated with co-expression network, the group of PKs is represented by 34% of the genes, even though the number of differentially expressed PP gene is slightly higher than that of PK genes (35 out of 205 PP genes and 173 out of 1,118 PK genes). Some modules contain only a few or none PP genes. For instance, the largest module contains only one PP gene, *PAP11*. These observations suggest that the regulation of biological processes may require a cascaded and/or coordinated protein phosphorylation by different PKs to adapt to environmental stresses, while the removal of phosphate from a phosphorylated protein by a PP is less specific. Because of the importance of PK in signaling and metabolism, it is reasonable to speculate that protein phosphorylation is one of the proprietary processes for plant cell to use the limited Pi under Pi deficiency.

### Genes involved in unidimensional cell growth form the major module

To gain insight into the function of the genes associated with the network, GO enrichment analyses were performed. The biological processes ‘glycolysis’, ‘negative regulation of anion channels activity by blue light’, ‘unidimensional cell growth’, and ‘glucosinolate biosynthetic process’ were most strongly enriched (Additional file 15). This analysis supports the assumption that protein phosphorylation is an important regulatory level for diverse processes associated with the recalibration of the cellular Pi homeostasis. It has been documented that Pi deficiency alters cellular metabolism, mainly by enhanced carbon flux via glycolysis for increased synthesis of organic acids [34]. Our data support these findings and further suggest that under Pi deficiency protein phosphorylation is an important regulator of glycolytic flux.

### Protein kinases involved in pollen tube development and growth are co-expressed in Pi-deficient roots

**Figure 3:**
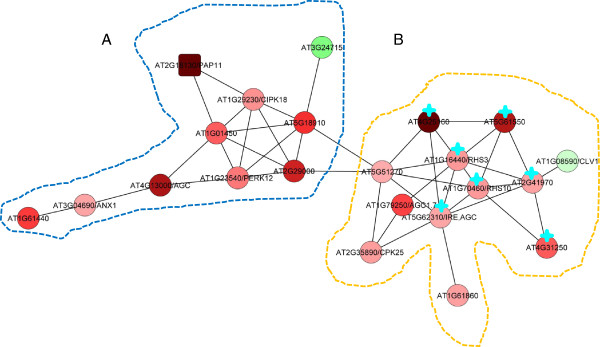
**Coexpression relationships of genes comprising PKPP1.** Red nodes indicate up-regulated genes; green nodes denote genes that are repressed by Pi deficiency. Round-shaped nodes represent protein kinase genes, rectangles indicate protein phosphatase genes. This network can be divided into two sub-clusters **A** and **B**. Green stars indicates root hair-related genes.

One of the robust changes in Arabidopsis roots under Pi deficiency is an increase in root hair length and density [9]. Several PK genes, including those that are related to root hair development, are differentially expressed under Pi deprivation and form the major module, indicating that protein phosphorylation is involved in this process. This module, named PKPP1 (PK and PP genes), consists of 21PK genes and one PP gene, PAP11 (Figure 3 and Table 1). More than 50% of the members in this module belong to the RLK superfamily, including seven RLKs (32%) and five RLCKs (Receptor-like cytoplasmic kinase, 23%). The second most abundant members are AGCs (cAMP dependent, cGMP dependent, and protein kinase C family, 18%), including two genes encoding proteins which were previously reported to be involved in root hair development and growth (list genes here) [35,36]. Other members in this module include CDPKs and genes for which no function has been assigned. Except two genes which were repressed by Pi deficiency, all other 20 genes were induced by Pi starvation (Table 1). Gene ontology analysis revealed that genes involved in the biological processes ‘unidimensional cell growth’, including ‘pollen tube growth’ were enriched in this module (Additional file 16). This module can further be divided into two sub-modules connected by a U-box domain-containing RLCK gene (At5g51270). The sub-module PKPP1A consists of nine PKs and one PP, PAP11 (Figure 3A). *PAP11* transcript was not detectable in roots under normal conditions but induced by Pi deficiency, although the transcript level was still low in Pi-deficient plants (Additional file 2). Notably, transcript of *PAP5*, the closest homologue of PAP11, was neither detectable in roots nor induced by Pi deficiency (Additional file 12). So far, experimental evidence for a possible biological function of PAP11 is lacking. *PAP11* was correlatively expressed with two PK genes in Pi-deficient roots, *At1g01450* and *CIPK18*, a CBL-interacting protein kinase (CIPK) superfamily protein which generally interacts with calcineurin B-like proteins (CBLs). It is thus reasonable to speculate that CIPK18 is be involved in the Pi deficiency-mediated signaling transduction via a calcium-dependent manner by interacting with AtCBL2 [37]. *CIPK18* was also correlatively expressed with three other RLK superfamily genes, *At2g29000* (LRR-1 subfamily), *At5g18190* (RLCK-VI subfamily), and *At1g23540*/*PERK12* (PERK (proline-rich extension-like receptor kinase) subfamily) [38-43]. RLKs are defined by the presence of a signal peptide, an extracellular domain (this domain is absent in the RLCK subfamily), a transmembrane domain region that anchors the receptor in cell membrane, and a carboxyl-terminal serine/threonine kinase domain [44]. More than 2% of the predicted Arabidopsis coding sequences encode RLKs, which have been associated with diverse functions in development, pathogen resistance, hormone perception, and environmental adaption [44]. Two RLKs, At4g29180 and At1g51880*,*also belonging to the LRR-1 subfamily, have been defined as *ROOT HAIR SPECIFIC* (*RHS*) genes *RHS16* and *RHS6* based on the presence of root hair element(RHE) consensus sequence in their promoter that drives root hair-specific gene expression [36,45,46]. *PERK12/IGI1*is highly expressed in anthers. A gain-of-function heterozygous mutant showed increased shoot branching and decreased plant height, while homozygous loss-of-function mutants were sterile with no inflorescence and an abnormal flower organ after the plants began to flower [47]. Further data showed that *PERK12* was down-regulated by auxin treatment, and the auxin efflux carrier *PIN1* and the auxin biosynthesis genes *CYP79B2* and *CYP79B3* showed altered expression levels in *igi1* mutants, linking PERK12 to auxin homeostasis [47]. This suggests a possible function of PERK12 in root development, especially in the altered lateral root and root hair development in response to Pi deficiency, since both processes are dependent on auxin movement between cells [48,49]. Another gene co-expressed with *PERK12* in Pi-deficient roots, *At4g13000,* belongs to the AGC VIII subfamily [50]. In yeast and mammals, AGCs have been implicated in the regulation of transcription, apoptosis, cell proliferation, insulin signaling, and cytoskeletal remodeling [51,52]. In plants, the signaling processes associated with AGC kinase activity and the mechanisms underlying the regulation of the kinase activity remain largely unknown. Some members of this family are involved in the regulation of auxin transport polarity, phototropin, planar growth, pollen tube and root hair growth [53], suggesting a possible involvement of At4g13000 in the Pi-deficiency response of the roots. The other gene co-expressed with *At4g13000* is *ANX1 (*At3g04690*)* which, together with its homologue*ANX2*, is involved in cell wall modification during pollen tube growth [54,55]. Interestingly, *ANX2* was neither detectable in roots under Pi-replete conditions nor induced by Pi deficiency. These data suggest that ANX2 might be functional mainly in pollen tube growth and is expressed in a tissue-specific manner, while ANX1 may be required for both pollen tube growth and root hair development. Notably, a gene encoding an S-locus lectin PK belonging to the SD-1 subfamily within the RLK superfamily, *At1g61440*, was co-expressed with *ANX1* under Pi deficiency [39]. At1g61440 is annotated with functions in recognition of pollen and carbohydrate binding. Experimental evidence for such functions remains experimental validation. *At3g24715* is the only gene that was down-regulated in response to Pi deficiency in this sub-module. Its product contains an octicosapeptide/phox/Bem1p domain, annotated with functions in reproduction and pollen tube development. In summary, most of the genes associated with sub-module PKPP1A are highly expressed in anthers and are mainly involved in the process of polarized cell growth in pollen tube development. Since most of these genes were induced by Pi deficiency, it is reasonable to assume that these genes are important for root-associated, Pi deficiency-induced developmental changes including root hair development and growth, but have minor impacto n root development and growth under normal conditions.

**Table 1:** Genes comprising PKPP1

**Locus**	**Annotation**	**Mean ± SD (−Pi/+Pi; P < 0.01)**
AT1G01450	Protein kinase superfamily protein	1.93 ± 0.92
AT1G23540	AtPERK12, IGI1, Protein kinase superfamily protein	1.60 ± 0.38
AT1G29230	ATCIPK18, ATWL1, CIPK18, SnRK3.20, WL1, CBL-interacting protein kinase 18	1.50 ± 0.45
AT1G61440	S-locus lectin protein kinase family protein	1.99 ± 0.51
AT2G29000	Leucine-rich repeat protein kinase family protein	3.03 ± 0.79
AT3G04690	ANX1, Malectin/receptor-like protein kinase family protein	1.36 ± 0.08
AT3G24715	Protein kinase superfamily protein with octicosapeptide/Phox/Bem1p domain	0.65 ± 0.03
AT4G13000	AGC (cAMP-dependent, cGMP-dependent and protein kinase C) kinase family protein	3.65 ± 0.62
AT5G18910	Protein kinase superfamily protein	2.19 ± 0.47
AT2G18130	ATPAP11, PAP11, purple acid phosphatase 11	∞ **(De novo synthesis)**
AT1G08590	Leucine-rich receptor-like protein kinase family protein	0.87 **±** 0.04
AT1G61860	Protein kinase superfamily protein	1.39 **±** 0.02
AT5G51270	U-box domain-containing protein kinase family protein	1.33 ± 0.13
AT5G61550	U-box domain-containing protein kinase family protein	3.52 ± 0.39
AT4G31250	Leucine-rich repeat protein kinase family protein	1.75 ± 0.12
AT4G25160	U-box domain-containing protein kinase family protein	6.13 ± 1.12
AT2G35890	CPK25, calcium-dependent protein kinase 25	1.39 ± 0.08
AT1G70460	RHS10, root hair specific 10	1.43 ± 0.18
AT1G79250	AGC1.7, AGC kinase 1.7	1.98 ± 0.44
AT5G62310	IRE, AGC (cAMP-dependent, cGMP-dependent and protein kinase C) kinase family protein	1.38 ± 0.25
AT2G41970	Protein kinase superfamily protein	1.38 ± 0.13
AT1G16440	RSH3, root hair specific 3	1.42 ± 0.17

Co-expression network PKPP1 (Protein Kinase Protein Phosphatase) is composed of 22 genes most of which are induced by Pi deficiency (P < 0.01).

### Protein kinases from the AGC and PERK subfamilies are critical for root hair development and growth under Pi deficiency

Members of sub-module PKPP1B (Figure 3B and Table 1) may play key roles in root hair development and elongation under both normal and Pi-deficient conditions. Three genes in this submodule, including the two root hair-specific genes *RHS3* and *RHS10*[36] and *IRE1* (INCOMPLETE ROOT HAIR ELONGATION 1) [35], have been related to root hair development and elongation. RHS3 and IRE1 belong to the AGC family while RSH10 belongs to the PERK family, indicating that kinases in these families are particularly important for root hair development and growth. All three genes were induced by Pi deficiency at the transcriptional level. Reverse genetic studies would be of help to decipher their physiological functions under Pi deprivation. Several other genes in this sub-module, including *At4g25160, At5g61550, At4g31250*, and *At2g41970*, have been reported to be part of a gene regulatory network comprising 208 root epidermal ‘core’ genes in Arabidopsis [45]. All genes in this sub-module were induced by Pi-deficiency, implicating their involvement in root hair development and growth under Pi starvation. AGC1.7, belonging to AGCVIII subfamily, and its homologue AGC1.5 have been reported to be critical for the polarized growth of pollen tubes [56], but have not yet been associated with root hair development. Our analysis revealed that *AGC1.7* was co-expressed with *RSH3* and *CPK25* (calcium-independent CDPK [57]) under Pi deficiency, suggesting that AGC1.7 supports a possible function in root hair development under Pi limiting conditions. The only gene that was repressed by Pi deficiency in the sub-module PKPP1B is *CLV1*. By binding to a small protein ligand CLV3, CLV1restricts the proliferation and/or promotes the differentiation of stem cells in the shoot apical meristem [58]. Interestingly, RNA-seq analysis revealed that *CLV1* was also highly expressed in Arabidopsis roots and decreased in response to Pi deficiency (Table 1), suggesting that CLV1 might be negatively regulating root hair development in response to Pi starvation.

### Root epidermial core gene associated with PKs and PPs under Pi deficiency

**Figure 4:**
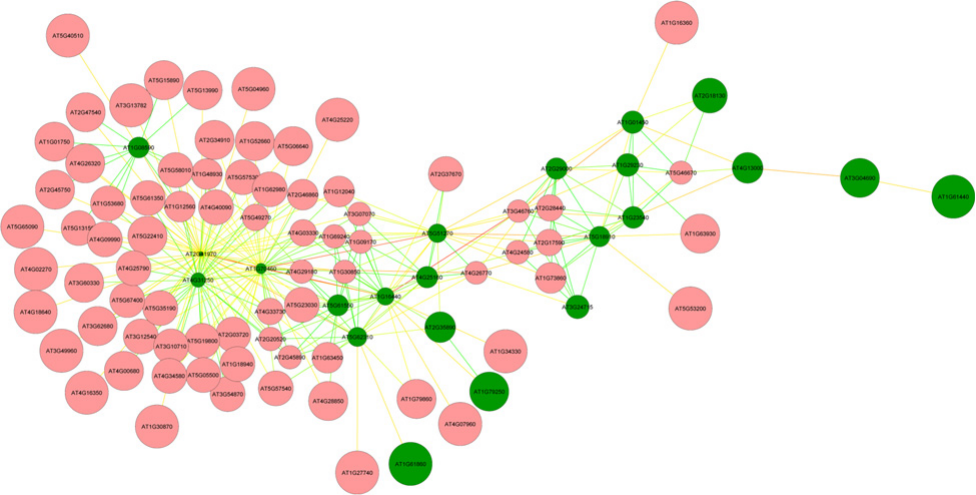
**Co expression relationships of genes comprising REPKPP1.** Red nodes indicate fished genes from the ‘core’ root hair genes, green nodes denote genes that derived from PKPP1. Node size corresponds to edge counts.

**Figure 5:**
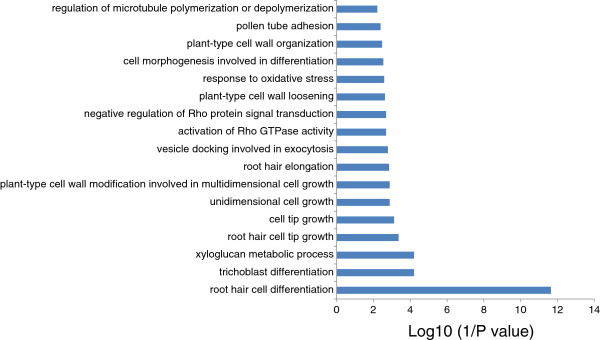
Gene ontology (GO) enrichment analysis of the genes from REPKPP1.

Several studies have shown that the expression of genes within the same metabolic pathway shows similar pattern; thus co-expression analysis can aid in discovering upstream regulators of a particular metabolic pathways [59,60]. For example, co-expression analysis identified MYB28 and MYB29 as regulators of aliphatic glucosinolate biosynthesis in Arabidopsis [61], and RSR1 as a regulator of starch biosynthesis in rice [62]. Further, CRC and AP1 have been identified as regulators of fatty acid biosynthesis in Arabidopsis in this way [63]. As described above, members of the major module are mainly involved in polarized cell growth, especially in root hair development and growth. To identify potential upstream regulators or downstream targets of the co-expressed PK and PP genes, the 208 root epidermal ‘core’ genes [45] were loaded together with 22 bait genes from module PKPP1 for subsequent co-expression analysis with a Pearson correlation coefficient cutoff of 0.7, resulting in a co-expression network, named REPKPP1 (root epidermal PK and PP genes), with 97 nodes and 279 edges (Figure 4 and Additional 17). Of the 75 genes that were fished from the root hair core genes, 46 were differentially expressed upon Pi deficiency (Additional file 18). Also this network can be divided into two sub clusters, the smaller of which contained much less edges and was composed of PKPP1A genes and six fished genes. Two of these six genes were induced by Pi deficiency; one of which (At163930) encodes the exocystinteractor ROH1 that has been associated with the localized deposition of seed coat pectin [64]. Gene ontology analysis revealed that these six genes are mainly involved in the biological processes of ‘mucilage biosynthetic process’, ‘trichome branching’, and ‘microtubule-based movement’ (Additional file 19). The larger subcluster consists of the PKPP1B and 69 fished genes, 44 of which were induced by Pi deficiency (Additional file 18). Gene ontology analysis uncovered that the 69 fished genes are chiefly involved in the biological processes of ‘root hair development and elongation’, ‘cell wall metabolic process’, ‘vesicle docking involved in exocytosis’, and ‘Rho GTPase activity’ (Figure 5 and Additional file 20). Most of the edges are mainly from four bait genes, *At1g70460, At2g41970, At4g31250*, and *At1g16440*, suggesting key roles of these genes in root hair development and growth under Pi deficiency. Of the four genes, *RSH10* (At1g70460) and *RSH3* (At1g16440), have been implicated in root hair development [36]; the function of the other two genes uncovered in the present study awaits to be experimentally addressed. Taken together, this network provides several potentially important regulatory PKs relevant to root hair development and growth under Pi starvation.

## Conclusions

In summary, we here provide precise digital information on the transcription of protein kinase and phosphatase genes in Arabidopsis roots at a genome-wide level. A root-specific co-expression network of Pi-responsive genes encoding protein kinases and phosphatases has been generated and putative novel players in Pi deficiency-induced root hair formation have been uncovered. Combined with the previously published root hair core genes [45], a comprehensive inventory for the regulation of root hair development and metabolism was obtained. The approach applied here will be useful to direct further studies by reverse genetic methods and eventually decipher the mechanisms by which root epidermal cells are re-programmed to adapt to Pi deficiency.

## Methods

### Data collection and processing

Transcriptome data of roots from plants grown in the presence or absence of Pi from 13-d-old Arabidopsis seedlings by RNA-seq were downloaded from a public database (NCBI: SRA050356.1) and analyzed as described in [22]. Microarray data of 2,671 ATH1 arrays from the NASCarray database (http://affymetrix.arabidopsis.info/) were downloaded and normalized using the RMA function of the Affy package of the Bioconductor software. Among the 2,671 arrays, 300 root-related arrays were manually identified as described in [9]. PK and PP genes were retrieved on the basis of TAIR 10 release of Arabidopsis genome.

### Generation of co-expression networks and modules of Pi-responsive PK and PP genes using the MACCU toolbox

To generate root-specific networks of Pi-responsive PK and PP genes, differentially expressed PK and PP genes in the Arabidopsis roots were obtained using a Student *t*-test at a P value <0.05. Gene networks were constructed based on 300 publicly available root-related arrays using the MACCU toolbox as described in [9], with a Pearson correlation threshold of 0.7. The generated co-expression networks were visualized by Cytoscape (http://www.cytoscape.org). If one cluster of genes did not have any connection (without any edges) to any other cluster in the co-expression network, we referred to such a cluster as a module.

### Construction of root hair-specific networks of Pi-responsive PK and PP genes

To obtain a root hair cell-specific network of Pi-responsive PK and PP genes, the 208 root epidermal ‘core’ genes were first mined from the datasheet described in [45]. Next, Pi-responsive PK and PP genes involved in the module of pollen tube/root hair development and growth were extracted (bait genes), combined with the genes from the root epidermal ‘core’ (preys), and used for generating co-expression network using the MACCU toolbox with a Pearson correlation threshold of 0.7. The resulting networks shows only those nodes (genes) and edges (relationships between genes) that were linked by at least one edge must with bait. Edges linked to two preys were excluded.

## Competing interests

We declare that we have no competing interests.

## Authors’ contributions

PL performed most of the work and drafted the manuscript. WL and WS participated in the analysis of the data. All authors approved the final version of the manuscript.

## Supplementary Material

Additional file 1 Expression of 1,118 kinase genes in Arabidopsis retrieved from TAIR10 roots. Click here for file

Additional file 2 Expression of 205 phosphatase genes in Arabidopsis roots retrieved from TAIR10. Click here for file

Additional file 3 Distribution of transcripts derived from protein kinase genes in Arabidopsis roots. Click here for file

Additional file 4 432 PK and 85 PP proteins identified in Arabidopsis roots. Click here for file

Additional file 5 Expression of the cysteine-rich RLK (receptor-like protein kinase) subfamily in Arabidopsis roots. Click here for file

Additional file 6 GO enrichment analysis of the 57 highly expressed protein kinase genes in Arabidopsis roots. Click here for file

Additional file 7 A subset of 29 protein phosphatase genes not or lowly expressed in Arabidopsis roots. Click here for file

Additional file 8 GO enrichment analysis of the subset of 29 protein phosphatase not or lowly expressed genes in Arabidopsis roots. Click here for file

Additional file 9 GO enrichment analysis of the subset of nine protein phosphatase genes with high abundance in Arabidopsis roots. Click here for file

Additional file 10 Subsets of 173 PK and 35 PP genes, comprising diverse subfamilies that were differentially expressed under Pi-deficient conditions in Arabidopsis roots. Click here for file

Additional file 11 GO enrichment analysis 205 protein kinase and phosphatase genes upon Pi deficiency in Arabidopsis roots. Click here for file

Additional file 12 Digital expression information of the 29 purple acid phosphatase genes in Arabidopsis roots. Click here for file

Additional file 13 **Co-expression relationships of protein kinase and phosphatase genes upon Pi deficiency in Arabidopsis roots.** Red nodes indicate up-regulated genes, green nodes denote genes that are repressed by Pi deficiency. Round-shaped nodes represent protein kinase genes, rectangles indicate protein phosphatase genes. Click here for file

Additional file 14 Genes comprising the PK/PP co-expression network in Arabidopsis roots. Click here for file

Additional file 15 GO enrichment analysis of 65 PK and PP genes involved in Co-expression network. Click here for file

Additional file 16 GO enrichment analysis of the 22 genes comprising the co-expression network PKPP1. Click here for file

Additional file 17 Protein-protein interaction pairs and statistics of edge enrichment for the co-expression network REPKPP1. Click here for file

Additional file 18 A subset of 75 Genes from the root epidermal ‘core’ genes fished for the co-expression network in Arabidopsis roots. Click here for file

Additional file 19 GO enrichment analysis of the six fished genes involved in the co-expression network REPKPP1A. Click here for file

Additional file 20 GO enrichment analysis of the 69 fished genes involved in the co-expression network REPKPP1B. Click here for file
