# Supplementary figures and images for: Genome-wide co-expression analysis predicts protein kinases as important regulators of phosphate deficiency-induced root hair remodeling in Arabidopsis
Source: BMC Genomics. 2013 Apr 1;14:210. doi: 10.1186/1471-2164-14-210 (PMC3636113; doi:10.1186/1471-2164-14-210)

## Slide 1
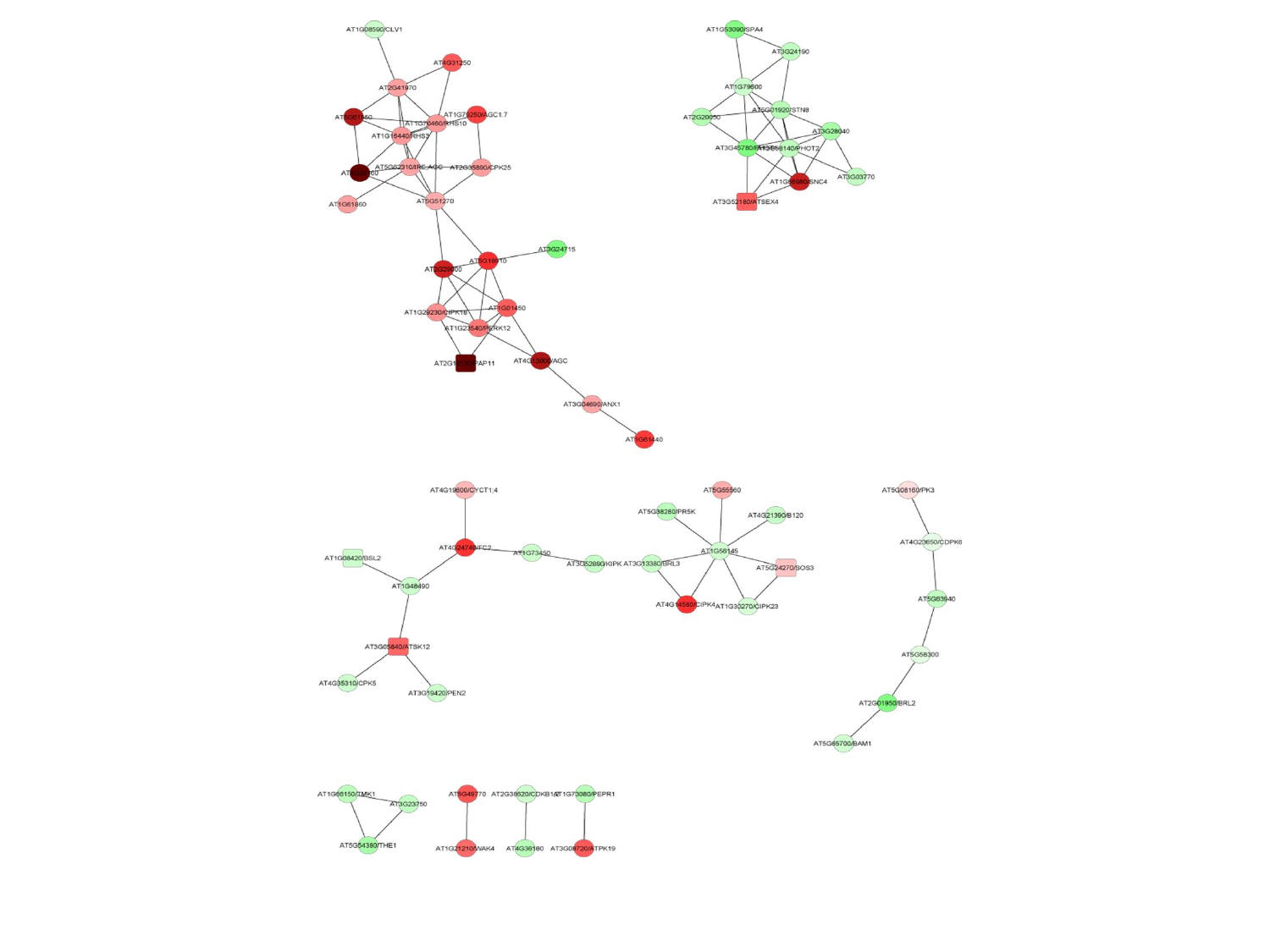

Supplement: Additional file 13 — Co-expression relationships of protein kinase and phosphatase genes upon Pi deficiency in Arabidopsis roots. Red nodes indicate up-regulated genes, green nodes denote genes that are repressed by Pi deficiency. Round-shaped nodes represent protein kinase genes, rectangles indicate protein phosphatase genes. [file 1471-2164-14-210-S13.pptx]
